# Dietary Fish Oil and a Flavor and Multi-Enzyme Complex Supplementation Improved the Reproductive Performance, Nutrient Metabolism and Health of Primiparous Lactating Sows and Piglets

**DOI:** 10.3390/ani16030379

**Published:** 2026-01-25

**Authors:** Lianpeng Zhao, Fangyuan Chen, Hu Zhang, Lingjie Huang, Liang Hu, Lun Hua, Lianqiang Che, Bin Feng, Yong Zhuo, Yan Lin, Shengyu Xu, De Wu, Pierre Cozannet, Simon Eskinazi, Zhengfeng Fang

**Affiliations:** 1 Key Laboratory for Animal Disease Resistance Nutrition of the Ministry of Education, Animal Nutrition Institute, Sichuan Agricultural University, Chengdu 611130, China; zlp9426@163.com (L.Z.); zhu68842@gmail.com (H.Z.); 71424@sicau.edu.cn (L.H.); hualun@sicau.edu.cn (L.H.); che.lianqiang@sicau.edu.cn (L.C.); fengbin@sicau.edu.cn (B.F.); zhuoyong@sicau.edu.cn (Y.Z.); linyan@sicau.edu.cn (Y.L.); shengyuxu@sicau.edu.cn (S.X.); wude@sicau.edu.cn (D.W.); 2 School of Food and Liquor Engineering, Sichuan University of Science and Engineering, Zigong 643000, China; c2023fangyuan@163.com; 3 Key Laboratory of Agricultural Product Processing and Nutrition Health (Co-Construction by Ministry and Province), College of Food Science, Sichuan Agricultural University, Ya’an 625014, China; huliang@sicau.edu.cn; 4 Adisseo France SAS, European Lab of Innovation, Science and Expertise, F-03600 Commentry, France; pierre.cozannet@adisseo.com; 5 Adisseo (Nutriad Ltd.) 1 Telford Court, Chester Gates, Chester CH1 6LT, UK; simon.eskinazi@adisseo.com

**Keywords:** fish oil, enzyme, flavor, sow, polyunsaturated fatty acid

## Abstract

This study was conducted to investigate the effects of dietary polyunsaturated fatty acid (PUFA) levels and FME (flavor and multiple enzymes) on the reproductive performance, nutrient digestion, and metabolism, immunity, and antioxidant capacity of sows and piglets. Forty primiparous sows [Duroc × (Landrace × York)] were randomly assigned from day 107 of gestation to day 7 post-weaning to one of four dietary treatments, low PUFA (4.6% tallow, LP), high PUFA (4.6% fish oil, HP), and LP and HP, each supplemented with 600 mg/kg FME (LP + FME, HP + FME). Results showed that dietary HP + FME supplementation significantly alleviated sow backfat loss during lactation (*p* < 0.05). Dietary FME supplementation significantly increased milk lactose and solids-non-fat (*p* < 0.05) on day 15. Meanwhile, milk protein and true protein contents were significantly lower in the LP treatment than in the LP + FME and HP treatments. The apparent total-tract digestibility (ATTD) of ash and phosphorus was improved (*p* < 0.05) by both HP diets and FME supplementation. The ATTD of energy and dry matter was significantly higher in LP + FME treatment than in LP and HP + FME treatments (*p* < 0.05). HP diets increased serum malondialdehyde (MDA, *p* < 0.01), total superoxide dismutase (*p* < 0.05) in sows, and increased serum MDA and decreased hydrogen peroxide (H_2_O_2_) contents in piglets (*p* < 0.05). Dietary FME supplementation decreased serum H_2_O_2_ contents and increased serum catalase activity of sows and/or piglets (*p* < 0.05). The serum immune markers, lipid, and protein metabolites of sows and piglets were altered (*p* < 0.05 or *p* < 0.10) by HP diets and/or FME supplementation. In conclusion, dietary fish oil (4.6% of diet replacing tallow) and FME (600 mg/kg) supplementation improved lactating performance by improving nutrient digestibility, body reserve mobilization, antioxidant capacity, and health state of sows and piglets.

## Simple Summary

During the critical transition period of farrowing and lactation, sows face a core physiological challenge: balancing energy demands, body condition loss, milk quality, and the immune requirements of both sows and offspring. Fish oil provides energy, reduces backfat loss, and—rich in omega-3 PUFAs—improves inflammation, lipid metabolism, milk quality, and immune status. However, high PUFA levels increase lipid peroxidation risk. FME (flavor and multi-enzyme additives) enhances feed intake, nutrient digestibility/utilization, and antioxidant capacity. This complementary strategy effectively resolves the conflict between energy deficit, body reserve mobilization, milk production, and immune needs in sows and piglets. This study investigated whether supplementing sow diets with fish oil and FME [flavor (proprietary mixture of aldehydes, ketones, and esters with red fruit and vanilla taste), multi-enzymes (xylanase, beta-glucanase, phytase)] could improve nutrient digestibility, antioxidant capacity, and reproductive performance. Results showed that sows fed fish oil with FME reduced backfat loss and produced higher-quality milk. Dietary fish oil and FME supplementation improved milk composition, nutrient digestibility, and serum metabolite (lipid, protein, immunity, and antioxidant capacity) profiles of sows and piglets, suggesting that fish oil and FME can help alleviate negative energy balance in lactating sows. These findings offer potential strategies for optimizing sow health and productivity.

## 1. Introduction

During farrowing, sows face a critical transition period as limited feed intake and the initiation of milk production require a large quantity of energy and nutrients (e.g., amino acids), which generally results in a decrease in sow body weight due to backfat and muscle mobilization [1]. The severe metabolic load during lactation also induces oxidative stress and inflammatory responses, and sows may not fully recover until weaning [2]. Excessive mobilization of body reserves has been linked to a prolonged weaning-to-estrus interval (WEI) and reduced farrowing rate [3]. Therefore, dietary strategies tailored to the nutritional needs of this period are crucial for optimizing milk yield, sow health, and reproductive performance [4,5].

Fish oil, a rich source of omega-3 polyunsaturated fatty acids (PUFAs), is widely recognized for its benefits in human health and reproduction [6,7], but relatively few relevant studies have been performed on livestock and poultry, especially pigs and sows. Omega-3 PUFAs act as ligands for transcription factors that regulate metabolism and development, and they influence inflammation and lipid metabolism [8]. Dietary PUFA supplementation has been associated with improved reproductive outcomes [9,10]. Moreover, supplementing sow diets with fish oil or seaweed extracts during late gestation and lactation improves piglet growth, fatty acid profiles, gut health, and maternal oxidative and immune status [11,12]. However, excessive PUFA intake can increase lipid peroxidation, and vitamin E supplementation alone is insufficient to counteract this effect [13].

Flavor additives are commonly used to stimulate feed intake and have been shown to improve sow intake and piglet growth [14]. In parallel, exogenous enzymes enhance nutrient digestibility, immune function, and antioxidant capacity in sows and pigs [15,16,17,18]. Multi-enzyme complexes are particularly effective and economical, as they improve digestion and nutrient utilization beyond the capacity of single enzymes. Nevertheless, high-PUFA diets may impair lipid digestion and elevate lipid peroxidation risk [19]. Recent evidence indicates that fiber-degrading and proteolytic enzymes promote lipid emulsification and absorption by reducing digesta viscosity and facilitating micelle formation [20,21]. Additionally, FME (flavor and multi-enzymes) may help improve the antioxidant system to mitigate high-level PUFA-induced lipid peroxidation.

Thus, combined supplementation of fish oil and FME may provide dual benefits for lactating sows, improving both nutrient metabolism and antioxidant capacity, thereby supporting growth and health in sows and piglets. We hypothesized that dietary fish oil and/or FME would enhance nutrient digestibility, feed intake, metabolism, and body reserve mobilization, ultimately benefiting sows and offspring. To test this hypothesis, primiparous sows were fed diets with and without supplementation of fish oil and/or FME from day 107 of gestation until day 7 post-weaning, and the potential role of diets in helping sows facing a negative energy balance was evaluated via growth performance, feed intake, milk yield and composition, nutrient digestibility, and serum metabolite (lipid, protein, immunity, and antioxidant capacity) profiles of sows and piglets.

## 2. Materials and Methods

### 2.1. Ethics Approval

The protocol of this study was approved (protocol code: 2022092114008 and date of approval: 21 September 2022) by the Animal Care and Use Committee of the Animal Nutrition Institute, Sichuan Agricultural University, and was carried out in accordance with the National Research Council’s Guide for the Care and Use of Laboratory Animals.

### 2.2. Experimental Design and Diets

A total of 40 crossbred [Duroc × (Landrace × York)] primiparous sows, selected from the same batch and artificially inseminated following synchronization of estrus, were assigned to four treatments, with 10 sows per treatment, on the basis of body weight (BW, 208.03 ± 1.87 kg) and backfat thickness (14.97 ± 0.26 mm) at 107 days of gestation. Sows were randomly assigned to receive one of the four diets. LP treatment: low-level PUFA diet (tallow); HP treatment: high-level PUFA diet (fish oil); LP + FME and HP + FME: LP or HP diets supplemented with a multi-enzyme (100 mg/kg of diet to supply at least 1250 U of xylanase, 860 U of beta-glucanase and 1000 FTU of phytase; Rovabio, Adisseo France SAS, France) and flavor (500 mg/kg; a proprietary chemically defined mixture of aldehydes, ketones, and esters, which have a taste of red fruit and vanilla; Krave, Adisseo (Nutriad Ltd. France SAS, Antony, France). This dosage was based on the recommendation from the product manufacturer and consistent with the validated effective dosage reported in our previous study [17]. Basal diets were formulated to meet or exceed the nutrient requirements of sows as recommended by the NRC (2012) (Table 1). Experimental diets were fed from 7 days before farrowing until 7 days after weaning (day 21 of lactation).

### 2.3. Animal Management

Oxytocin was injected on day 114 of gestation for synchronal parturition. The BW and backfat thickness of sows and the BW of piglets were measured individually within 48 h after farrowing. Cross-fostering was conducted within each dietary treatment to balance the litter size and average litter weight. Sows were reared individually in temperature-controlled farrowing crates with free access to water. Two sows (LP, HP + FME) were removed from the study due to poor health and low feed intake during the experimental period, and three sows (LP, LP + FME and HP + FME) were excluded because of postpartum paralysis.

The feed regimen is shown in Supplementary Figure S1. Briefly, 2.5 kg was provided on days 107–110 of gestation, and then 2 kg was provided on days 111–113 of gestation; no feed was provided on the farrowing day. A total of 2 kg was provided on day 1 of lactation, and then the feed allowance was increased by 1 kg/d until day 7 after farrowing, after which feed was offered ad libitum. To allow maximal feed intake potential, feed troughs were filled with sufficient feed to ensure that there was feed left in the trough by the time of the next feeding. Sows were fed four times per day (at 08:00, 12:00, 16:00, and 20:00). In addition, to collect fasting blood samples during the experiment, four 12 h fasts (from 20:00 to 08:00) were imposed. Subsequently, to monitor the WEI, sows were offered 3.5 kg of feed per day from day 21 of lactation (weaning) until the end of the experiment on day 28 of lactation.

### 2.4. Data and Sample Collection

Sow BW and backfat thickness were measured after fasting on day 107 of gestation and within 24 h after farrowing and at weaning. The thickness of backfat was measured at P2 position (65 mm from the backbone at the position of the last rib bone, both on the right and left sides of the sow). Daily feed intake of sows was recorded. The BW of piglets was measured on days 1, 7, 14, and 21 to evaluate average daily weight gain (ADG).

Diet samples were collected according to each distribution, summed into sample bags, and stored at −20 °C. Fecal samples were collected from sows on days 18–20 of lactation via manual rectal stimulation, with two separate sampling time points (09:00 and 17:00) for each sow. The fresh fecal samples were mixed, and a few drops of toluene and hydrochloric acid (10% fresh fecal sample) were added for preservation and nitrogen volatilization prevention. The samples were stored frozen at −20 °C for later determination of apparent total tract digestibility of nutrients.

Blood samples were collected from sows (ear vein) at day 107 of gestation and on days 1, 7, 15, and 22 of lactation after 12 h fasting. Piglet blood was collected on days 1 and 22 (one piglet/litter). The serum was separated by centrifugation at 3000 revolutions per minute (rpm) and 4 °C for 15 min; the supernatant was aliquoted and stored at −20 °C until analysis. Colostrum was sampled from the anterior four teats within 1 h of farrowing. On days 15 and 22, after blood sampling, 1 mL of oxytocin was injected (ear vein) to facilitate milk collection.

### 2.5. Analysis of Fecal and Dietary Samples

All the diets and feces were oven-dried (65 °C) to a constant weight for analyses of dry matter (DM), gross energy (GE), CP, ash, and Ca and P contents according to the AOAC (2007). Gross energy was measured via an adiabatic oxygen bomb calorimeter (PARR 6400, Parr Instruments Company, Moline, IL, USA). The acid-insoluble ash was analyzed according to previous methods [22] and was used as a marker to calculate the apparent total tract digestibility (ATTD):

$$Digestibility\;(\%)=100-\frac{Indicator\;in\;feed\;(\%)}{Indicator\;in\;feces\;(\%)}\times \frac{Components\;content\;in\;feces\;(\%)}{Components\;content\;in\;feed\;(\%)}\times 100$$

Dietary total fatty acids were trans-esterified via a previously reported method with modifications [23]. Briefly, approximately 200 mg of a freeze-dried dietary sample was weighed into a 10 mL glass test tube, and 3 mL of a chloroform–methanol–water mixture with a volume ratio of 8:4:3 was added. The mixture was subjected to vortex oscillation for 1 min, ice bath ultrasonication for 5 min, repeated five times, and then incubated at 4 °C for 2 h. Afterwards, the mixture was centrifuged at 1500 rpm for 15 min. The next step involved separating the chloroform layer into another 10 mL glass tube, washing the remaining residue with 3 mL of a chloroform–methanol–water (8:4:3) mixture twice, then transferring all the obtained chloroform layer to the above glass tube; the fatty acid glyceride mixture was obtained by drying in a vacuum drying oven. Then, 2 mol of 0.5 mol KOH-CH_3_OH was added to the reaction tube and sealed, and the mixture was reacted in a water bath at 50 °C for 10 min. The mixture was then cooled to room temperature, and 2 mL BF_3_-CH_3_OH (w = 14%) was added. After the tube was covered and oscillated for 10 s, it was heated in a water bath at 80 °C for 2 min to complete the methyl ester. Then, 1 mL of hexane (0.05 g/L BHT n-hexane solution) and 2 mL of saturated sodium chloride were added for full-swirl oscillation extraction. The upper n-hexane layer (not less than 600 μL) was separated into the vapor phase machine vial to be tested. Finally, fatty acid methyl esters were analyzed via a SHIMADZU GC-2010 Plus gas chromatograph with a flame ionization detector (Shimadzu, Kyoto, Japan). Fatty acids are distinguished according to the length of the carbon chain and the number and location of the double bonds.

### 2.6. Analysis of Blood and Milk Samples

Serum creatinine, triglycerides (TG), non-esterified fatty acids (NEFA), total cholesterol (TC), HDL-cholesterol (HDL-C), LDL-cholesterol (LDL-C), aspartate amino-transferase (AST), alanine aminotransferase (ALT), gamma-glutamyl transpeptidase (GGT), alkaline phosphatase (ALP), C-reactive protein (CRP), and IgG and IgM were measured by using a 3100 automatic biochemical analyzer (Hitachi, Tokyo, Japan) according to the manufacturer’s procedure.

Serum-free amino acids were determined according to methods described in a previous study [24]. Briefly, 300 μL of a serum sample was mixed with 900 μL of 10% sulfosalicylic acid (1:3), left for 30 min at 4 °C, and then centrifuged at 12,000× *g* and 4 °C for 15 min. The supernatant was filtered through a 0.22 μm-pore-size PTFE syringe filter (Millipore, Cork, Ireland) into an autosampler vial, and the amino acid content was measured via an automatic amino acid analyzer LA8080 (Hitachi, Tokyo, Japan). Amino acid standard solutions of type B and AN-II (Wako Pure Chemical Industries Ltd., Osaka, Japan) were used for calibration.

The activities of total-superoxide dismutase (T-SOD), catalase (CAT), and glutathione peroxidase (GSH-Px), and the levels of malondialdehyde (MDA), total antioxidant capacity (T-AOC), oxidized glutathione (GSSH), total glutathione (GSH), H_2_O_2_, and protein carbonyl were measured via kits (Nanjing Jiancheng Bioengineering Institute, Nanjing, China). The serum content of haptoglobin (HP-globin) was measured by using an HP-globin ELISA kit (Ruixinbio Ltd., Quanzhou, China) according to the manufacturer’s procedure. The contents of fat, protein, lactose, DM, urea nitrogen, and solids-non-fat in colostrum and milk samples were analyzed via a Combifoss FT+ analyzer (Foss Electric, Hillerød, Denmark).

### 2.7. Statistical Analysis

The statistical analysis was performed via the PROC MIXED procedure of SAS 9.4 software (Cary, NC, USA). Unless otherwise specified, data, including reproductive performance, milk composition, and digestibility coefficients, were analyzed in a factorial treatment design with a randomized complete design via the following models:

Yijk = µ + αi + βj + (αβ) ij + eijk

where Y (Yijk) is an observed trait, µ is the population mean, αi is the main effect of the diets (i = LP, HP), βj is the main effect of FME supplementation (j = −FME, +FME), (αβ)ij is the interaction between diets and FME supplementation, and e (eijk) is the residual, which was assumed to be normally distributed and to have variance homogeneity.

The repeated-measures data, including the serum oxidative status, parameters, and amino acid composition of sows and piglets, were analyzed via the MIXED procedure according to the following model:

Yijk = μ + ai + βi + rk + (αβ)ij + (αγ)ik + (βγ)jk + (αβγ)ijk + εijk

where Y (Yijk) is an observed trait, µ is the population mean, αi is the main effect of the diet (i = LP, HP), βj is the main effect of FME supplementation (j = −FME, +FME), γk is the effect of time (k  =  1, 2, 3 or 4), (αγ)ik is the interaction between the diet and time, (βγ)jk is the interaction between FME supplementation and time, (αβγ)ijk refers to the interaction among the diet, FME supplementation and time, and εijk represents the residual error. The sows and the litters represented the experimental units. The means were obtained via the LSMEANS method. When an interaction between the factors was significant, a pairwise comparison was made via the Adjust = Tukey option in SAS. The results were considered significantly different for *p* < 0.05 and tended toward 0.05 ≤ *p* < 0.10. SEM = standard error of the mean.

## 3. Results

### 3.1. Effects of PUFA Levels and FME Supplementation on Reproductive Performance of Sows

The backfat loss of sows was affected (*p* < 0.05) by the diet × FME interaction during lactation (Table 2). Compared with the other treatments, dietary HP + FME significantly reduced sow backfat loss during lactation. The total (*p* = 0.086) and average (*p* = 0.085) milk yields tended to be increased by dietary FME supplementation regardless of dietary PUFA levels. Daily feed intake of lactating sows was influenced by the passage of time (Supplementary Figure S2).

### 3.2. Effects of PUFA Levels and FME Supplementation on Composition of Milk Samples

Compared with the LP treatment, dietary HP supplementation tended to decrease colostrum fat content (*p* = 0.072, Table 3). The protein and true protein contents in milk on lactation day 15 were affected by the diet × FME interaction (*p* < 0.05). Specifically, protein and true protein contents in milk were significantly lower in the LP group compared with the LP + FME and HP groups. Compared with the diets without FME supplementation, dietary FME supplementation increased the lactose (*p* < 0.01), dry matter (*p* = 0.089), and solids-non-fat (*p* < 0.01) contents in milk on day 15 of lactation. Moreover, the content of lactose (day 22) was significantly decreased by dietary HP supplementation among these treatments.

### 3.3. Effects of PUFA Levels and FME Supplementation on Digestibility Coefficients of Sows

**Figure 1:**
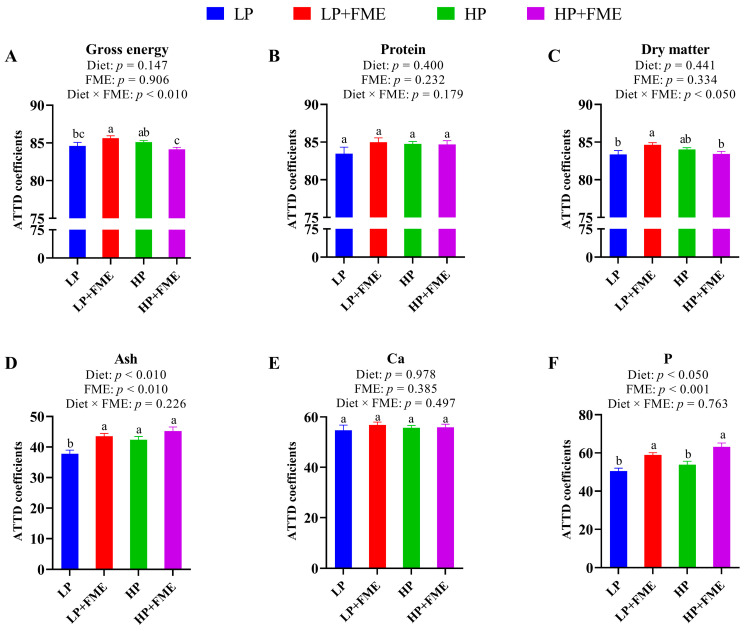
Effects of PUFA levels and FME supplementation on apparent total-tract digestibility of nutrients in sows during lactation. (**A**), ATTD of gross energy; (**B**), ATTD of protein; (**C**), ATTD of dry matter; (**D**), ATTD of ash; (**E**), ATTD of Ca; (**F**), ATTD of P. ATTD = apparent total-tract digestibility; P = phosphorus; Ca = calcium. ^a,b,c^ means with no common letters differ at *p* < 0.05 (Tukey’s post hoc test following significant diet × FME interaction).

The ATTD of GE and DM of sows was significantly affected (*p* < 0.05) by the diet × FME interaction (Figure 1). Notably, the ATTD of GE and DM in sows were significantly higher in the LP + FME treatment than in LP and HP + FME treatments (*p* < 0.05), whereas no significant difference was observed between LP + FME and HP treatments. High PUFA levels and FME supplementation both improved (*p* < 0.05) ATTD of ash and P. Interestingly, the ATTD of P increased by 6.8% and 17.0% with high PUFA levels and FME supplementation, respectively.

### 3.4. Effects of PUFA Levels and FME Supplementation on Serum Oxidative Status of Sows

Compared with the diets without FME supplementation, dietary FME supplementation decreased serum H_2_O_2_ levels (*p* < 0.01) and increased CAT activity (*p* < 0.05) in sows (Table 4). Compared with LP diets, HP diets elevated average serum T-SOD (*p* < 0.05), MDA (*p* < 0.01), and T-AOC (*p* = 0.072) levels of sows. The time effect indicated that serum protein carbonyl, H_2_O_2_, CAT, T-SOD, T-AOC, and GSSG/T-GSH ratio changed with the progression of lactation (*p* < 0.05 or *p* < 0.01). Specifically, as lactation progressed, the serum protein carbonyl, H_2_O_2_, and T-AOC levels gradually increased, whereas the CAT, T-SOD, and GSSG/T-GSH ratio decreased in sows.

### 3.5. Effects of PUFA Levels and FME Supplementation on Serum Oxidative Status of Piglets

Compared with the diets without FME supplementation, dietary FME supplementation increased serum CAT (*p* < 0.01, Table 5) activity in piglets. Compared with LP diets, HP diets elevated serum MDA (*p* < 0.05), T-SOD (*p* < 0.05), and decreased H_2_O_2_ (*p* < 0.05) content in piglets. On day 0 of lactation, the serum protein carbonyl content and GSSG/T-GSH ratio were significantly greater than those on day 22 of lactation (*p* < 0.05), whereas serum MDA, H_2_O_2_, T-SOD, and T-AOC contents were significantly lower than those on day 22 (*p* < 0.05). Moreover, the serum MDA content of piglets was affected by the diet × time interaction (*p* < 0.05). On day 22 of lactation, piglets from sows fed HP diets presented a significant increase in serum MDA content (*p* < 0.05).

### 3.6. Effects of PUFA Levels and FME Supplementation on Serum Parameters of Sows

Serum GGT activity was affected by the diet × FME × time interaction (*p* < 0.05, Table 6), with the highest activity observed in HP + FME sows on day 22. The serum HDL-C levels were greater in the LP treatment than those in the HP and HP + FME treatments (*p* < 0.05). Moreover, serum HDL-C, CRP, ALP, AST/ALT, IgM, and IgG levels were affected (*p* < 0.05) by the diet × time or FME × time interaction. Compared with the diets without FME supplementation, dietary FME supplementation decreased serum ALT activities (*p* < 0.01) and increased IgG (*p* < 0.05) content in sows. Compared with LP diets, HP diets elevated serum ALP (*p* < 0.01) and GGT (*p* < 0.05) and decreased average HDL-C (*p* < 0.01) and CRP (*p* < 0.05) contents in sows. In addition, as lactation progressed, the serum TC, TG, HDL-C, LDL-C, NEFA, and IgG levels gradually increased from days 0 to 15 of lactation (*p* < 0.05), whereas at day 22, the levels of these indicators were lower than those at day 15. The serum ALT and CREA levels of sows on day 0 of lactation were the highest throughout the whole stage (*p* < 0.05), whereas the serum ALP levels on day 7 of lactation were the lowest (*p* < 0.05).

### 3.7. Effects of PUFA Levels and FME Supplementation on Serum Parameters of Piglets

Compared with the diets without FME supplementation, dietary FME supplementation increased serum Hp-globin (*p* < 0.01), HDL-C, ALP, AST, ALT, and AST/ALT (*p* < 0.05) levels in piglets (Table 7). Compared with LP diets, HP diets elevated serum IgM (*p* < 0.05) and decreased TG (*p* < 0.01) content in piglets. In addition, on day 0 of lactation, the serum ALP, GGT, CREA, and IgG contents were significantly greater than those on day 22 of lactation (*p* < 0.05), whereas the serum TC, TG, HDL-C, LDL-C, CRP, Hp-globin, ALT, NEFA, and IgG contents were significantly lower than those on day 22 (*p* < 0.05). Moreover, serum HDL-C, CRP (*p* = 0.084), ALP, and AST/ALT ratio of piglets were affected (*p* < 0.05) by the diet × time or FME × time interaction.

### 3.8. Effects of PUFA Levels and FME Supplementation on Serum Amino Acid Composition of Sows

**Figure 2:**
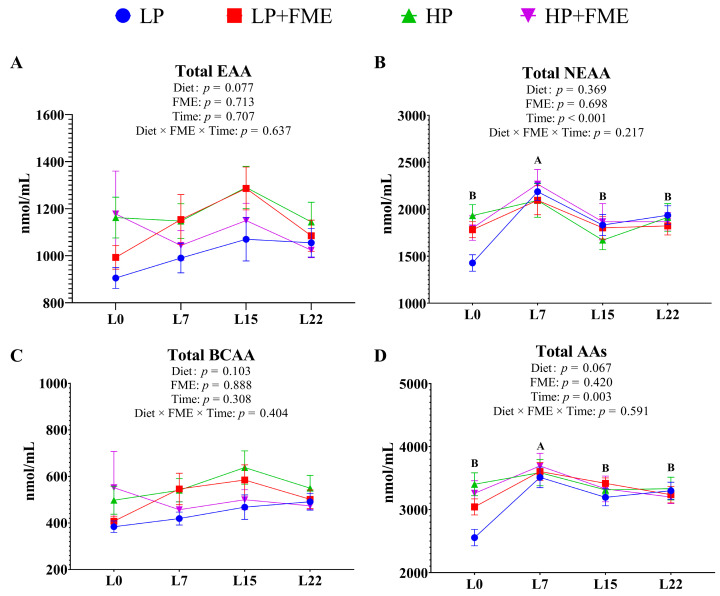
Effects of PUFA levels and FME supplementation on the serum amino acid composition of sows. (**A**), total essential amino acid, total EAA; (**B**), total non-essential amino acid, total NEAA; (**C**), total branched-chain amino acid, total BCAA; (**D**), total amino acids, total AAs. L0 = Day 0 of lactation; L7 = Day 7 of lactation; L15 = Day 15 of lactation; L22 = Day 22 of lactation. ^A,B,^ (time) means with no common letters differ at *p* < 0.05.

Compared with LP diets, HP diets tended to increase the serum essential amino acids (EAA, *p* = 0.077) and total amino acids (AAs, *p* = 0.067) concentrations in sows (Figure 2 and Supplementary Table S1). Additionally, serum non-essential amino acids (NEAA) and total AAs concentrations of sows on day 7 of lactation were significantly lower than those at other time points (*p* < 0.01).

### 3.9. Effects of PUFA Levels and FME Supplementation on Serum Amino Acid Composition of Piglets

**Figure 3:**
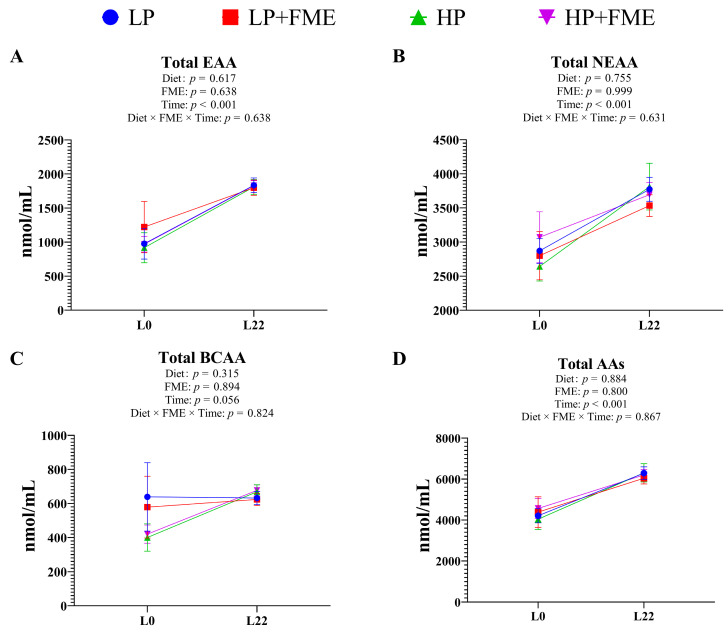
Effects of PUFA levels and FME supplementation on the serum amino acid composition of piglets. (**A**), total essential amino acid, total EAA; (**B**), total non-essential amino acid, total NEAA; (**C**), total branched-chain amino acid, total BCAA; (**D**), total amino acids, total AAs. PD0: piglets on day 0 of lactation; PD22: piglets on day 22 of lactation.

The serum EAA, NEAA, branched-chain amino acids (BCAA), and total AAs concentrations in piglets were not affected by FME supplementation or PUFA levels (Figure 3 and Supplementary Table S2). Furthermore, the time effect indicated that serum EAA, NEAA, and total AAs concentrations of weaning piglets were significantly greater than in newborn piglets (*p* < 0.01).

## 4. Discussion

The delivery and perinatal periods impose a sharp increase in energy and nutrient demands for sows while voluntary feed intake is often limited. This mismatch commonly triggers mobilization of body reserves and can compromise postpartum recovery, subsequent reproductive performance, and piglet health [25,26]. Therefore, this study aimed to evaluate the effects of dietary PUFA levels and FME supplementation on the reproductive performance, nutrient digestion, body mobilization, immunity, and antioxidant capacity of sows and their offspring.

In this study, high PUFA levels were shown to decrease backfat loss in sows during lactation. FME supplementation decreased backfat loss in sows by 50%, although the difference was not statistically significant. The lack of statistical significance may be attributed to limited sample size and substantial inter-individual variability in backfat measurements, influenced by baseline body condition and individual feed intake [27,28]. Similarly, these findings are consistent with reports that n − 3 PUFA and exogenous enzymes improve lipid metabolism and nutrient utilization [15,29,30], respectively, thereby helping to preserve sow body condition during the energetically demanding lactation period. Notably, fish oil and FME synergistically reduced backfat loss in sows more effectively than either intervention alone, supporting our hypothesis that their synergy improves sow body condition during the high-energy demands of lactation. In addition, FME tended to increase milk yield and significantly enhanced milk lactose, DM, and solids-non-fat, aligning with recent evidence that multi-enzyme (cellulase, xylanase, β-glucanase, and β-mannanase) supplementation significantly improves milk yield and composition via enzymatic hydrolysis of feed ingredients [31,32]. Overall, the enhanced digestive and absorptive capacity conferred by FME, along with the metabolic reprogramming mediated by PUFA, may collectively contribute to the improvement of milk composition and the enhancement of milk production potential.

The mechanisms underlying the observed improvements in milk composition and yield are likely multifactorial. First, the flavor component (proprietary mixture of aldehydes, ketones, and esters imparting red fruit and vanilla notes) may have enhanced feed palatability and intake, indirectly supporting milk production and body condition maintenance [14,17]. However, the lack of a significant effect on ADFI in this study may reflect feeding management and restrictions [33,34] due to four 12 h fasting periods for blood sampling during the experiment. Second, dietary PUFA and FME increased ATTD of ash and phosphorus, consistent with previous reports [16,35,36]. GE and DM digestibility showed a diet × FME interaction with the LP + FME group outperforming LP and HP + FME. These patterns suggest selective effects of lipid profile and enzymes on nutrient fractions, likely mediated by improved intestinal integrity, microbial composition, and enzyme–substrate interactions [36,37]. The multi-enzyme complex in FME (primarily xylanase, β-glucanase, and phytase) contributed to these improvements by degrading non-starch polysaccharides and phytate, reducing digesta viscosity, and enhancing nutrient utilization [18,20,21]. Better digestibility helps explain the improved milk yield and milk composition we observed. Although these benefits did not translate into short-term increases in litter weight or ADG of piglets, they may bolster offspring health and resilience [38]. This is supported by our previous study, where piglets from LP + FME and HP + FME treatments, when challenged with LPS after weaning, exhibited enhanced resistance to LPS-induced inflammation as a result of maternal fish oil supplementation [39].

Lactating sows experience systemic oxidative stress due to increased metabolic demands, resulting in a transient decline in antioxidant capacity that resolves only in late lactation [2]. In the present study, temporal changes in serum H_2_O_2_, CAT, T-SOD, and T-AOC levels in sows confirmed the occurrence of oxidative stress during lactation, and antioxidant enzyme activities were significantly higher in weaned piglets than in neonates. In addition, a previous study reported that multi-enzymes (acid protease, fungal α-amylase, xylanase, β-mannanase, glucose oxidase, acid cellulose, galactosidase) increased antioxidant enzyme activity and decreased peroxide products by improving nutrient digestibility and utilization [18]. Our study demonstrated that FME reduced serum H_2_O_2_ levels and increased CAT activity in both sows and piglets. Additionally, high PUFA intake increased serum MDA in sows and piglets, with a more pronounced effect in piglets during late lactation, while concurrently elevating T-SOD activity and T-AOC and reducing H_2_O_2_ levels. These findings support the dual nature of PUFAs: susceptibility to lipid peroxidation yet activation of protective antioxidant and anti-inflammatory pathways [40,41,42,43]. High PUFA increased serum IgM and decreased CRP, while FME increased IgG and Hp-globin. CRP rises during lactation as an acute-phase inflammatory marker [17]. HP-globin binds free hemoglobin to exert antioxidant and immunomodulatory effects [44]. Collectively, PUFA and FME exerted complementary actions on innate and adaptive immunity, improving oxidative–immune balance in lactating sows and their offspring.

In our study, high PUFA affected lipid profiles differently in sows and piglets (e.g., reductions in TG and changes in HDL-C) and increased GGT activity, alongside modest increases in ALP, and AST/ALT. Moreover, the time effects of these lipid metabolites represent significant metabolic changes in sows during lactation. The synthesis of lipids and cholesterol in the body may increase dramatically in the early stages of lactation to meet the fat and cholesterol requirements of milk. n−3 PUFAs significantly reduce serum TC, TG, and LDL-C concentrations, which are recognized as major risk factors for cardiovascular disease [45]. However, evidence remains inconsistent regarding whether high PUFA intake improves HDL-C levels [46]. GGT cleaves glutathione to maintain intracellular redox balance, thereby preventing oxidative stress-induced apoptosis [47]. Importantly, AST/ALT ratios remained within physiological limits [48], suggesting heightened hepatic metabolic activity rather than overt hepatocellular damage. Newborn piglets showed higher baseline liver enzyme levels that likely reflect developmental physiology rather than pathology. ALP not only is an indicator of liver function but also plays an important role in bone formation [49,50,51]. The marked increase in neonatal ALP with maternal FME may relate to altered hepatic–biliary handling or bone metabolism in early life.

High dietary PUFA tended to increase maternal serum essential and total amino acids, indicating improved amino acid availability or altered utilization. This supports the notion that PUFAs can influence amino acid transport and metabolic signaling, thereby affecting maternal protein and milk synthesis [52,53,54]. Interestingly, we found that the serum concentrations of most AAs were significantly improved by high dietary PUFA levels on the day of delivery and throughout lactation. This may be due to the contribution of PUFAs to the metabolism and immunity of sows in response to childbirth stress and abnormal nutrient metabolism during lactation. In addition, this study has several limitations. The use of a commercial FME product prevented separation of flavor and multi-enzyme effects. Future studies may develop personalized multi-enzyme flavor systems by matching enzymes to diet-specific anti-nutritional factors and using flavor agents to improve palatability, enabling synergistic enzymatic enhancement and flavor optimization.

## 5. Conclusions

Supplementation of 4.6% fish oil plus 600 mg/kg FME in primiparous sows from late gestation to lactation effectively alleviated lactational negative energy balance, reduced backfat loss, improved milk composition, boosted nutrient digestibility, and enhanced maternal antioxidant and immune function, thus improving reproductive performance and metabolic health. Additionally, this nutritional strategy exerted maternal transfer effects to strengthen piglets’ antioxidant capacity and immunity, benefiting their growth. These findings provide theoretical and practical support for its application in large-scale swine production.

## Figures and Tables

**Table 1:** Levels of ingredients in the diets.

		LP Diet	HP Diet
Raw material composition, %			
	Wheat	20.0	20.0
	Corn	43.9	43.9
	Soybean meal	7.5	7.5
	Rapeseed meal	19.1	19.1
	Tallow	4.6	0.0
	Fish oil	0.0	4.6
	DL-Met hydroxy analog	0.1	0.1
	L-Lysine hydrochloride (98%)	0.4	0.4
	L-threonine (98.5%)	0.1	0.1
	Limestone	0.2	0.2
	Calcium hydrogen phosphate	2.2	2.2
	Sodium chloride	0.4	0.4
	Premix ^a^	1.5	1.5
Chemical composition, %			
	Protein	16.2	16.2
	Starch	35.7	35.7
	Fat	7.0	7.0
	Net energy, MJ/kg	10.53	10.53
Digestible amino acids, %			
	Arginine	0.82	0.82
	Histidine	0.36	0.36
	Isoleucine	0.53	0.53
	Leucine	1.12	1.12
	Lysine	0.92	0.92
	Methionine	0.31	0.31
	Methionine + cysteine	0.48	0.48
	Phenylalanine	0.61	0.61
	Phenylalanine + tyrosine	1.06	1.06
	Threonine	0.58	0.58
	Tryptophan	0.18	0.18
	Valine	0.63	0.63
Minerals, %			
	Calcium	0.90	0.90
	Digestible Phosphorus	0.50	0.50
Fatty acid profiles, analysis value (g/kg feed)			
	C18:2*n* − 6	10.50	10.95
	C18:3*n* − 3	0.75	1.39
	C20:3*n* − 3	0.01	0.13
	C20:5*n* − 3	0.15	7.27
	C22:6*n* − 3	0.08	5.77
	∑ *n* − 6 ^b^	10.50	10.95
	∑ *n* − 3 ^c^	0.99	14.56
	PUFA ^d^	11.49	25.51

^a^ Provided per kg of diet: vitamin A, 6000 IU; vitamin D_3_, 1200 IU; vitamin E, 50 IU; menadione, 2.4 mg; thiamine, 1 mg; riboflavin, 3.6 mg; niacin, 20 mg; d-panthothenic acid, 12.5 mg; vitamin B_6_, 1.8 mg; vitamin B_12_, 12.5 μg; d-biotin, 240 μg; folic acid, 2 mg; copper, 25 mg; iron, 100 mg; manganese, 35 mg; zinc, 125 mg; iodine, 0.20 mg; selenium, 0.3 mg; anti-mold (sodium propionate), 0.5 g; mycotoxin adsorbent (montmorillonoid), 1 g; Antioxidants (ethoxyquin and propionate gallate), 0.2 g. ^b^ ∑ *n* − 6: C18:2*n* − 6; ^c^ ∑ *n* − 3: C18:3*n* − 3, C20:3*n* − 3, C20:5*n* − 3, C22:6*n* − 3; ^d^ PUFAs = ∑ *n* − 6 + ∑ *n* − 3.

**Table 2:** Effects of PUFA levels and FME supplementation on reproductive performance of sows during lactation.

Item	Treatment				SEM	Diet		FME		*p*-Value		
	LP	LP + FME	HP	HP + FME		LP	HP	−FME	+FME	Diet	FME	Diet × FME
Sows, n	8	9	10	8								
Body weight loss, kg	−4.94	−5.61	−7.05	−1.69	2.54	−5.27	−4.37	−5.99	−3.65	0.724	0.363	0.243
Backfat loss, mm	−0.99 ^a^	−1.16 ^a^	−0.99 ^a^	0.33 ^b^	0.34	−1.07	−0.33	−0.99	−0.41	0.038	0.102	0.039
ADFI, kg	5.08	5.38	5.30	5.88	0.32	5.23	5.59	5.19	5.63	0.264	0.184	0.662
Total milk yield, kg	156.37	160.85	152.25	160.41	3.57	158.61	156.33	154.31	160.63	0.527	0.086	0.609
Average milk yield, kg/d	7.45	7.66	7.25	7.64	0.17	7.55	7.44	7.35	7.65	0.525	0.085	0.604
WEI, d	5.17	5.25	6.00	4.75	0.49	5.21	5.38	5.58	5.00	0.738	0.250	0.191
Litter size, n												
Initial (after fostering)	9	9	9	9								
Weaning	8.5	8.78	8.40	8.63	0.32	8.64	8.51	8.45	8.70	0.551	0.240	0.901
Sucking piglet weight, kg												
Day 1	1.48	1.48	1.49	1.49	<0.01	1.48	1.49	1.48	1.49	0.041	0.301	0.923
Day 7	2.77	2.71	2.74	2.75	0.08	2.74	2.75	2.76	2.73	0.949	0.753	0.647
Day 14	4.58	4.54	4.27	4.47	0.15	4.56	4.37	4.43	4.50	0.212	0.638	0.424
Day 21	6.44	6.46	6.04	6.41	0.24	6.45	6.22	6.24	6.43	0.340	0.421	0.453
ADG, g												
Day 1–7	184.13	175.31	176.65	180.71	11.61	179.72	178.68	180.39	178.01	0.929	0.839	0.583
Day 7–14	235.85	249.31	209.36	226.69	14.90	242.58	218.03	222.60	238.00	0.110	0.310	0.898
Day 14–21	265.47	274.44	228.35	277.31	18.62	269.96	252.83	246.91	275.88	0.365	0.130	0.291
Day 1–21	231.77	234.76	210.55	230.92	11.37	233.27	230.89	221.31	232.84	0.285	0.318	0.459

ADFI = average daily feed intake; WEI = weaning-to-estrus interval; ADG = average daily gain. n = X replicates per treatment. ^a,b^ Means within a row with no common letters differ at *p* < 0.05 or *p* < 0.01 (Tukey’s post hoc test following significant diet × FME interaction).

**Table 3:** Effects of PUFA levels and FME supplementation on milk composition of sows during lactation.

Item	Treatment				SEM	Diet		FME		*p*-Value		
	LP	LP + FME	HP	HP + FME		LP	HP	−FME	+FME	Diet	FME	Diet × FME
Colostrum												
Fat, %	5.79	6.11	5.07	4.90	0.52	5.95	4.99	5.43	5.51	0.072	0.884	0.637
Protein, %	15.67	15.05	16.09	16.46	0.87	15.36	16.27	15.88	15.75	0.305	0.884	0.577
True protein, %	14.66	14.10	15.09	15.46	0.85	14.38	15.28	14.88	14.78	0.302	0.908	0.589
Lactose, %	3.13	3.01	3.02	2.84	0.15	3.07	2.93	3.08	2.93	0.378	0.323	0.866
Dry matter, %	29.00	28.24	28.48	28.45	1.15	28.62	28.46	28.74	28.34	0.892	0.734	0.750
Solids-non-fat, %	23.18	22.03	23.38	23.50	0.86	22.61	23.44	23.28	22.77	0.340	0.554	0.467
Urea nitrogen (mg/dL)	56.50	56.06	59.02	51.56	2.45	56.28	55.29	57.76	53.81	0.690	0.117	0.161
Milk (L15)												
Fat, %	8.62	11.01	9.77	9.73	1.04	9.81	9.75	9.20	10.37	0.953	0.267	0.252
Protein, %	5.28 ^b^	6.38 ^a^	6.25 ^a^	5.95 ^ab^	0.29	5.83	6.10	5.76	6.17	0.355	0.179	0.023
True protein, %	4.39 ^b^	5.38 ^a^	5.32 ^a^	5.04 ^ab^	0.27	4.89	5.18	4.86	5.21	0.277	0.193	0.025
Lactose, %	5.38	5.78	4.87	5.83	0.24	5.58	5.35	5.12	5.80	0.355	0.009	0.247
Dry matter, %	22.41	26.52	24.07	24.64	1.33	24.46	24.35	23.24	25.58	0.935	0.089	0.193
Solids-non-fat, %	14.01	15.56	14.61	15.18	0.37	14.78	14.9	14.31	15.37	0.761	0.007	0.188
Urea nitrogen (mg/dL)	49.05	55.63	57.58	53.31	4.04	52.34	55.44	53.31	54.47	0.449	0.776	0.189
Milk (L22)												
Fat, %	6.35	6.76	6.5	6.16	0.42	6.55	6.33	6.42	6.46	0.594	0.932	0.377
Protein, %	4.65	4.83	5.17	4.80	0.18	4.74	4.99	4.91	4.82	0.176	0.613	0.128
True protein, %	3.87	4.01	4.73	4.01	0.17	3.94	4.19	4.12	4.01	0.142	0.530	0.145
Lactose, %	5.65 ^a^	5.76 ^a^	5.28 ^b^	5.84 ^a^	0.08	5.70	5.56	5.47	5.80	0.087	<0.001	0.001
Dry matter, %	19.42	20.17	19.71	19.50	0.55	19.79	19.60	19.56	19.83	0.734	0.628	0.393
Solids-non-fat, %	13.39	13.70	13.57	13.69	0.20	13.55	13.63	13.48	13.69	0.685	0.304	0.656
Urea nitrogen (mg/dL)	38.95	42.69	44.77	41.30	2.09	40.82	43.03	41.86	42.00	0.297	0.948	0.095

L15 = Day 15 of lactation; L22 = Day 22 of lactation. ^a,b^ Means within a row with no common letters differ at *p* < 0.05 or *p* < 0.01 (Tukey’s post hoc test following significant diet × FME interaction).

**Table 4:** Effects of PUFA levels and FME supplementation on the serum oxidative status of sows.

Item	Treatment				*p*-Value						
	LP	LP + FME	HP	HP + FME	Diet	FME	Time	Diet × FME	Diet × Time	FME × Time	Diet × FME × Time
Protein carbonyl, nmol/mg prot					0.135	0.421	<0.001	0.459	0.533	0.239	0.111
L0	0.19 ± 0.03	0.16 ± 0.03	0.16 ± 0.03	0.17 ± 0.03			C				
L7	0.24 ± 0.03	0.28 ± 0.03	0.26 ± 0.03	0.25 ± 0.03			B				
L15	0.25 ± 0.04	0.23 ± 0.03	0.18 ± 0.03	0.21 ± 0.04			BC				
L22	0.35 ± 0.03	0.35 ± 0.03	0.37 ± 0.03	0.23 ± 0.03			A				
Malondialdehyde (MDA), nmol/mL					<0.001	0.101	0.310	0.629	0.312	0.387	0.622
L0	3.23 ± 0.53	2.99 ± 0.50	3.06 ± 0.48	3.96 ± 0.53							
L7	2.99 ± 0.51	1.82 ± 0.48	4.22 ± 0.46	3.51 ± 0.51							
L15	2.88 ± 0.68	2.85 ± 0.64	3.96 ± 0.61	3.02 ± 0.68							
L22	3.48 ± 0.47	2.69 ± 0.44	4.79 ± 0.42	4.32 ± 0.47							
Hydrogen peroxide (H_2_O_2_), nmol/L					0.278	<0.001	<0.001	0.563	0.619	0.075	0.788
L0	30.65 ± 3.70	25.64 ± 3.49	29.14 ± 3.31	29.86 ± 3.70			B				
L7	54.40 ± 4.93	32.46 ± 4.64	44.82 ± 4.41	32.42 ± 4.93			A				
L15	47.94 ± 4.48	41.77 ± 4.23	43.82 ± 4.01	35.78 ± 4.48			A				
L22	45.88 ± 6.71	33.38 ± 6.33	43.65 ± 6.01	29.83 ± 6.71			AB				
Catalase (CAT), U/mL					0.324	0.038	<0.001	0.385	0.772	0.119	0.511
L0	11.88 ± 2.42	12.93 ± 2.28	10.30 ± 2.17	14.36 ± 2.42			A				
L7	14.33 ± 2.45	16.13 ± 2.31	13.94 ± 2.19	18.32 ± 2.45			A				
L15	8.87 ± 2.51	16.13 ± 2.36	12.49 ± 2.24	17.66 ± 2.51			A				
L22	8.70 ± 1.45	5.47 ± 1.37	7.66 ± 1.30	10.06 ± 1.45			B				
Total-superoxide dismutase (T-SOD), U/mL					0.037	0.657	<0.001	0.571	0.657	0.068	0.108
L0	159.56 ± 5.34	170.75 ± 5.03	162.27 ± 4.77	166.74 ± 5.34			A				
L7	134.88 ± 6.47	134.57 ± 6.27	164.66 ± 5.79	141.35 ± 6.47			B				
L15	102.70 ± 8.53	113.58 ± 8.04	108.56 ± 7.63	114.61 ± 8.53			C				
L22	148.94 ± 6.65	139.59 ± 6.27	144.13 ± 5.96	155.39 ± 6.65			B				
Total antioxidant capacity (T-AOC), U/L					0.072	0.305	0.014	0.552	0.966	0.395	0.793
L0	1.36 ± 0.32	1.46 ± 0.30	1.58 ± 0.29	1.64 ± 0.032			AB				
L7	1.40 ± 0.19	1.15 ± 0.18	1.57 ± 0.17	1.61 ± 0.19			B				
L15	1.13 ± 0.21	1.53 ± 0.20	1.37 ± 0.19	1.66 ± 0.21			B				
L22	1.86 ± 0.31	1.83 ± 0.29	1.88 ± 0.28	2.37 ± 0.31			A				
GSSG/T-GSH					0.356	0.103	<0.001	0.033	0.800	0.979	0.017
L0	0.42 ± 0.03 ^a^	0.38 ± 0.03 ^a^	0.38 ± 0.03 ^a^	0.34 ± 0.03 ^a^			A				
L7	0.35 ± 0.03 ^a^	0.29 ± 0.03 ^abc^	0.29 ± 0.03 ^ab^	0.31 ± 0.01 ^ab^			B				
L15	0.43 ± 0.03 ^a^	0.28 ± 0.03 ^abcd^	0.29 ± 0.03 ^ab^	0.40 ± 0.03 ^a^			AB				
L22	0.11 ± 0.03 ^d^	0.12 ± 0.03 ^cd^	0.15 ± 0.03 ^bcd^	0.10 ± 0.03 ^d^			C				

L0 = Day 0 of lactation; L7 = Day 7 of lactation; L15 = Day 15 of lactation; L22 = Day 22 of lactation. ^a,b,c,d^ (Tukey’s post hoc test following significant diet × FME × time interaction) or A, B, C (time) means within a row with no common letters differ at *p* < 0.05 or *p* < 0.01.

**Table 5:** Effects of PUFA levels and FME supplementation on the serum oxidative status of piglets.

Item	Treatment				*p*-Value						
	LP	LP + FME	HP	HP + FME	Diet	FME	Time	Diet × FME	Diet × Time	FME × Time	Diet × FME × Time
Protein carbonyl, nmol/mg prot					0.462	0.324	<0.001	0.082	0.974	0.363	0.088
PD0	0.73 ± 0.08	0.96 ± 0.07	0.81 ± 0.07	0.67 ± 0.08			A				
PD22	0.23 ± 0.08	0.23 ± 0.07	0.29 ± 0.07	0.26 ± 0.08			B				
Malondialdehyde (MDA), nmol/mL					0.029	0.897	0.005	0.925	<0.001	0.170	0.391
PD0	3.49 ± 0.41	4.00 ± 0.38	3.21 ± 0.36	3.40 ± 0.41			B				
PD22	3.52 ± 0.41	3.03 ± 0.38	5.13 ± 0.36	5.08 ± 0.41			A				
Hydrogen peroxide (H_2_O_2_), nmol/L					0.002	0.168	<0.001	0.154	0.756	0.802	0.359
PD0	57.17 ± 7.99	52.55 ± 7.53	38.53 ± 7.15	27.13 ± 7.99			B				
PD22	102.41 ± 12.61	107.74 ± 11.89	92.85 ± 11.28	64.05 ± 12.61			A				
Catalase (CAT), U/mL					0.176	0.005	0.440	0.562	0.490	0.723	0.403
PD0	7.97 ± 2.02	10.77 ± 1.90	7.06 ± 1.80	13.75 ± 2.02							
PD22	7.43 ± 2.02	11.53 ± 1.90	10.61 ± 1.80	14.12 ± 2.02							
Total-superoxide dismutase (T-SOD), U/mL					0.029	0.577	<0.001	0.258	0.913	0.807	0.111
PD0	113.40 ± 8.85	100.74 ± 8.35	111.90 ± 7.92	128.24 ± 8.85			B				
PD22	131.16 ± 6.36	137.43 ± 6.00	145.01 ± 5.69	147.36 ± 6.36			A				
Total antioxidant capacity (T-AOC), U/L					0.148	0.282	0.011	0.812	0.896	0.874	0.776
PD0	1.01 ± 0.28	1.12 ± 0.27	1.27 ± 0.25	1.62 ± 0.28			B				
PD22	1.55 ± 0.39	1.85 ± 0.36	1.88 ± 0.35	2.17 ± 0.39			A				
GSSG/T-GSH					0.534	0.915	<0.001	0.420	0.889	0.302	0.962
PD0	0.24 ± 0.02	0.25 ± 0.02	0.22 ± 0.02	0.25 ± 0.02			B				
PD22	0.20 ± 0.02	0.18 ± 0.02	0.18 ± 0.01	0.18 ± 0.02			A				

PD0: piglets on day 0 of lactation; PD22: piglets on day 22 of lactation. A, B (time) means within a row with no common letters differ at *p* < 0.05 or *p* < 0.01.

**Table 6:** Effects of PUFA levels and FME supplementation on the serum parameters of sows.

Item	Treatment				*p*-Value						
	LP	LP + FME	HP	HP + FME	Diet	FME	Time	Diet × FME	Diet × Time	FME × Time	Diet × FME × Time
Total cholesterol (TC), mmol/L					0.325	0.674	<0.001	0.082	0.009	0.021	0.133
L0	1.50 ± 0.10	1.35 ± 0.09	1.27 ± 0.09	1.34 ± 0.10			C				
L7	1.95 ± 0.10	1.86 ± 0.10	1.68 ± 0.09	1.51 ± 0.10			B				
L15	2.04 ± 0.14	1.89 ± 0.13	2.05 ± 0.12	2.27 ± 0.14			A				
L22	1.83 ± 0.11	1.83 ± 0.10	1.52 ± 0.09	2.02 ± 0.11			B				
Triglycerides (TG), mmol/L					0.370	0.986	<0.001	0.678	0.975	0.844	0.724
L0	0.18 ± 0.04	0.24 ± 0.04	0.20 ± 0.04	0.20 ± 0.04			B				
L7	0.21 ± 0.04	0.26 ± 0.04	0.21 ± 0.04	0.19 ± 0.04			B				
L15	0.39 ± 0.04	0.34 ± 0.04	0.35 ± 0.04	0.36 ± 0.04			A				
L22	0.27 ± 0.04	0.25 ± 0.04	0.25 ± 0.04	0.23 ± 0.04			B				
HDL-cholesterol (HDL-C), mmol/L					<0.001	0.520	<0.001	0.023	0.018	0.467	0.297
L0	0.45 ± 0.05	0.48 ± 0.04	0.41 ± 0.04	0.44 ± 0.05			C				
L7	0.79 ± 0.04	0.71 ± 0.04	0.54 ± 0.04	0.54 ± 0.04			B				
L15	0.84 ± 0.05	0.77 ± 0.04	0.62 ± 0.04	0.76 ± 0.04			A				
L22	0.84 ± 0.04	0.78 ± 0.04	0.57 ± 0.04	0.71 ± 0.04			A				
LDL-cholesterol (LDL-C), mmol/L					0.533	0.958	<0.001	0.058	0.353	0.229	0.165
L0	0.76 ± 0.06	0.68 ± 0.06	0.68 ± 0.06	0.73 ± 0.06			C				
L7	0.91 ± 0.06	0.90 ± 0.06	0.92 ± 0.06	0.79 ± 0.06			B				
L15	1.05 ± 0.06	0.94 ± 0.06	1.05 ± 0.06	1.15 ± 0.06			A				
L22	0.85 ± 0.06	0.84 ± 0.06	0.76 ± 0.06	0.99 ± 0.06			B				
C-reactive protein (CRP), mg/L					0.045	0.206	<0.001	0.469	0.692	<0.001	0.412
L0	9.69 ± 0.86	10.39 ± 0.82	8.11 ± 0.77	8.84 ± 0.86			AB				
L7	10.79 ± 0.92	10.32 ± 0.87	8.96 ± 0.83	10.64 ± 0.92			A				
L15	8.48 ± 0.72	9.71 ± 0.67	7.63 ± 0.64	9.78 ± 0.72			B				
L22	8.59 ± 0.43	8.11 ± 0.41	7.88 ± 0.39	6.89 ± 0.43			C				
Haptoglobin (Hp-globin), μg/mL					0.529	0.517	0.862	0.485	0.829	0.858	0.786
L0	188.63 ± 44.36	181.76 ± 44.14	183.41 ± 44.09	222.99 ± 44.36							
L7	195.09 ± 69.47	192.44 ± 69.21	192.85 ± 65.39	274.83 ± 69.47							
L15	242.89 ± 71.17	215.91 ± 70.92	211.88 ± 75.25	237.50 ± 71.17							
L22	175.61 ± 70.05	200.40 ± 66.05	219.84 ± 65.75	223.54 ± 70.05							
Alkaline phosphatase (ALP), mmol/L					<0.001	0.120	<0.001	0.848	0.018	0.178	0.267
L0	33.38 ± 4.36	36.78 ± 4.11	41.00 ± 3.90	45.38 ± 4.36			A				
L7	23.13 ± 4.39	26.67 ± 4.14	33.20 ± 3.93	30.13 ± 4.39			B				
L15	28.25 ± 3.73	32.67 ± 3.52	40.60 ± 3.34	48.63 ± 3.73			A				
L22	24.88 ± 3.77	28.78 ± 3.56	38.60 ± 3.37	48.75 ± 3.77			A				
Gamma-glutamyl transpeptidase (GGT), U/L					0.049	0.800	0.141	0.806	0.065	0.001	0.030
L0	35.51 ± 4.22 ^ab^	34.34 ± 3.98 ^ab^	46.85 ± 3.77 ^ab^	33.99 ± 4.22 ^b^							
L7	36.96 ± 4.01 ^ab^	36.76 ± 3.78 ^ab^	45.85 ± 3.58 ^ab^	33.67 ± 4.01 ^b^							
L15	35.65 ± 4.77 ^ab^	38.40 ± 4.50 ^ab^	43.28 ± 4.27 ^ab^	46.35 ± 4.77 ^b^							
L22	33.65 ± 6.36 ^ab^	39.72 ± 6.00 ^ab^	39.85 ± 5.69 ^ab^	61.94 ± 6.36 ^a^							
Aspartate amino-transferase (AST), U/L					0.576	0.202	0.067	0.920	0.004	0.327	0.725
L0	25.62 ± 3.67	28.39 ± 3.46	21.78 ± 3.28	20.77 ± 3.67							
L7	21.32 ± 3.15	20.63 ± 2.97	19.43 ± 2.82	18.72 ± 3.15							
L15	16.79 ± 1.86	19.75 ± 1.75	21.54 ± 1.66	28.61 ± 1.86							
L22	16.71 ± 1.40	18.75 ± 1.32	20.42 ± 1.25	23.34 ± 1.40							
Alanine amino-transferase (ALT), U/L					0.786	0.001	<0.001	0.373	0.008	0.145	0.621
L0	35.42 ± 1.49	37.17 ± 1.41	33.88 ± 1.34	36.79 ± 1.49			A				
L7	32.85 ± 1.41	33.86 ± 1.33	31.66 ± 1.26	31.66 ± 1.41			BC				
L15	29.54 ± 0.78	31.01 ± 0.74	30.68 ± 0.70	34.63 ± 0.78			C				
L22	30.88 ± 1.08	33.94 ± 1.02	30.74 ± 0.97	36.14 ± 1.08			B				
AST/ALT					0.401	0.704	0.202	0.808	0.023	0.539	0.869
L0	0.72 ± 0.07	0.75 ± 0.07	0.64 ± 0.07	0.58 ± 0.07							
L7	0.64 ± 0.07	0.61 ± 0.07	0.61 ± 0.07	0.59 ± 0.07							
L15	0.57 ± 0.07	0.64 ± 0.07	0.70 ± 0.07	0.83 ± 0.07							
L22	0.54 ± 0.07	0.55 ± 0.07	0.66 ± 0.07	0.65 ± 0.07							
Creatinine (CREA), μmol/L					0.209	0.832	<0.001	0.787	0.032	0.011	0.428
L0	180.63 ± 11.01	190.49 ± 10.38	190.26 ± 9.85	179.95 ± 11.01			A				
L7	134.01 ± 6.17	120.49 ± 5.82	130.47 ± 5.52	123.87 ± 6.17			B				
L15	117.37 ± 6.53	115.98 ± 6.16	129.38 ± 5.84	134.88 ± 6.53			B				
L22	114.41 ± 6.52	118.37 ± 6.14	114.84 ± 5.83	135.21 ± 6.52			B				
Non-esterified fatty acids (NEFA), mmol/L					0.977	0.987	<0.001	0.950	0.999	0.924	0.827
L0	466.94 ± 82.37	386.59 ± 77.66	436.56 ± 77.57	409.15 ± 82.37			B				
L7	274.16 ± 133.56	352.48 ± 125.92	310.15 ± 119.46	287.84 ± 133.56			B				
L15	821.18 ± 199.36	935.66 ± 187.95	878.06 ± 178.31	857.24 ± 199.36			A				
L22	589.71 ± 180.75	460.98 ± 170.41	495.57 ± 161.67	593.49 ± 180.75			B				
IgM, g/L					0.721	0.137	<0.001	0.130	0.016	0.119	0.163
L0	1.47 ± 0.12	1.46 ± 0.11	1.17 ± 0.11	1.53 ± 0.12			A				
L7	1.40 ± 0.08	1.41 ± 0.08	1.26 ± 0.07	1.31 ± 0.08			A				
L15	1.26 ± 0.08	1.16 ± 0.07	1.24 ± 0.07	1.40 ± 0.08			A				
L22	1.09 ± 0.07	1.17 ± 0.07	1.04 ± 0.06	1.27 ± 0.07			B				
IgG, g/L					0.456	0.046	<0.001	0.066	0.259	<0.001	0.868
L0	3.46 ± 0.29	3.26 ± 0.27	3.19 ± 0.26	3.61 ± 0.29			C				
L7	4.16 ± 0.26	4.18 ± 0.25	3.78 ± 0.23	4.49 ± 0.26			B				
L15	4.48 ± 0.25	4.28 ± 0.23	4.28 ± 0.22	4.89 ± 0.25			A				
L22	3.85 ± 0.20	4.36 ± 0.19	3.96 ± 0.18	4.99 ± 0.20			B				

L0 = Day 0 of lactation; L7 = Day 7 of lactation; L15 = Day 15 of lactation; L22 = Day 22 of lactation. ^a,b,c^ (Tukey’s post hoc test following significant diet × FME × time interaction) or A, B, C (time) means within a row with no common letters differ at *p* < 0.05 or *p* < 0.01.

**Table 7:** Effects of PUFA levels and FME supplementation on the serum parameters of piglets.

Item	Treatment				*p*-Value						
	LP	LP + FME	HP	HP + FME	Diet	FME	Time	Diet × FME	Diet × Time	FME × Time	Diet × FME × Time
Total cholesterol (TC), mmol/L					0.376	0.961	<0.001	0.329	0.180	0.304	0.306
PD0	0.58 ± 0.08	0.73 ± 0.07	0.65 ± 0.07	0.84 ± 0.08			B				
PD22	2.96 ± 0.35	3.11 ± 0.33	2.91 ± 0.31	2.38 ± 0.35			A				
Triglycerides (TG), mmol/L					0.006	0.269	<0.001	0.067	0.151	0.671	0.051
PD0	0.20 ± 0.07	0.29 ± 0.06	0.12 ± 0.06	0.18 ± 0.07			B				
PD22	0.77 ± 0.07	0.98 ± 0.06	0.73 ± 0.06	0.60 ± 0.07			A				
HDL-cholesterol (HDL-C), mmol/L					0.068	0.047	<0.001	0.441	<0.001	0.218	0.105
PD0	0.23 ± 0.04	0.30 ± 0.04	0.27 ± 0.04	0.40 ± 0.04			B				
PD22	1.16 ± 0.04	1.26 ± 0.04	1.06 ± 0.04	1.00 ± 0.08			A				
LDL-cholesterol (LDL-C), mmol/L					0.337	0.891	<0.001	0.269	0.324	0.523	0.340
PD0	0.35 ± 0.04	0.43 ± 0.04	0.37 ± 0.04	0.42 ± 0.04			B				
PD22	1.65 ± 0.02	1.80 ± 0.02	1.66 ± 0.02	1.32 ± 0.02			A				
C-reactive protein (CRP), mg/L					0.263	0.142	<0.001	0.457	0.324	0.084	0.734
PD0	3.63 ± 0.12	3.71 ± 0.11	3.71 ± 0.11	3.67 ± 0.12			B				
PD22	4.30 ± 0.20	4.80 ± 0.18	4.68 ± 0.18	4.91 ± 0.20			A				
Haptoglobin (Hp-globin), μg/mL					0.766	0.002	0.020	0.972	0.500	0.155	0.678
PD0	443.28 ± 24.60	516.64 ± 23.19	463.67 ± 22.00	513.68 ± 24.60			B				
PD22	496.45 ± 50.90	622.61 ± 47.99	460.87 ± 45.53	606.24 ± 50.90			A				
Alkaline phosphatase (ALP), mmol/L					0.749	0.010	<0.001	0.338	0.900	0.029	0.194
PD0	1186.38 ± 219.49	1489.33 ± 206.94	987.3 ± 196.32	1788.38 ± 219.49			A				
PD22	412.63 ± 59.09	514.33 ± 55.71	463.00 ± 52.85	511.38 ± 59.09			B				
Gamma-glutamyl transpeptidase (GGT), U/L					0.409	0.513	<0.001	0.872	0.179	0.116	0.839
PD0	52.22 ± 5.06	54.65 ± 4.77	57.84 ± 4.52	60.52 ± 5.06			A				
PD22	37.60 ± 1.86	32.63 ± 1.75	37.08 ± 1.66	30.20 ± 1.86			B				
Aspartate amino-transferase (AST), U/L					0.520	0.039	0.592	0.527	0.185	0.036	0.857
PD0	18.02 ± 9.03	39.77 ± 8.52	10.73 ± 8.08	28.57 ± 9.03							
PD22	23.64 ± 2.88	26.47 ± 2.71	30.84 ± 2.58	26.33 ± 2.88							
Alanine amino-transferase (ALT), U/L					0.998	0.028	<0.001	0.927	0.446	0.099	0.288
PD0	21.37 ± 1.71	24.15 ± 1.61	21.00 ± 1.53	25.95 ± 1.71			B				
PD22	33.35 ± 1.03	34.98 ± 0.97	33.55 ± 0.92	33.37 ± 1.03			A				
AST/ALT					0.326	0.042	0.231	0.638	0.120	0.040	0.913
PD0	0.84 ± 0.30	1.51 ± 0.28	0.50 ± 0.27	1.14 ± 0.30							
PD22	0.71 ± 0.07	0.76 ± 0.07	0.90 ± 0.07	0.79 ± 0.07							
Creatinine (CREA), μmol/L					0.377	0.801	<0.001	0.721	0.754	0.626	0.759
PD0	98.50 ± 17.30	99.23 ± 16.31	102.87 ± 15.48	114.80 ± 17.30			A				
PD22	47.83 ± 2.49	45.07 ± 2.35	51.78 ± 2.22	49.70 ± 2.49			B				
Non-esterified fatty acids (NEFA), mmol/L					0.407	0.920	<0.001	0.611	0.779	0.616	0.863
PD0	47.81 ± 11.53	45.93 ± 10.87	20.91 ± 10.32	39.46 ± 11.53			B				
PD22	247.44 ± 27.65	236.67 ± 26.07	232.79 ± 24.73	233.11 ± 27.64			A				
IgM, mg/L					0.049	0.382	<0.001	0.166	0.027	0.921	0.098
PD0	7.88 ± 15.33	39.44 ± 14.46	5.40 ± 13.71	9.71 ± 16.39			B				
PD22	287.25 ± 49.63	230.89 ± 46.80	319.10 ± 44.39	422.29 ± 53.06			A				
IgG, g/L					0.131	0.265	<0.001	0.790	0.655	0.755	0.108
PD0	2.83 ± 0.14	3.29 ± 0.13	3.34 ± 0.13	3.17 ± 0.14			A				
PD22	1.87 ± 0.31	1.90 ± 0.29	1.99 ± 0.28	2.45 ± 0.31			B				

PD0: piglets on day 0 of lactation; PD22: piglets on day 22 of lactation. A, B (time) means within a row with no common letters differ at *p* < 0.05 or *p* < 0.01.

## Data Availability

None of the data were deposited in an official repository. Data that support the present study are available upon request.

## Supplementary Materials

The following supporting information can be downloaded at: https://www.mdpi.com/article/10.3390/ani16030379/s1.
