# Supplementary material for: Dietary Fish Oil and a Flavor and Multi-Enzyme Complex Supplementation Improved the Reproductive Performance, Nutrient Metabolism and Health of Primiparous Lactating Sows and Piglets
Source: Animals (Basel). 2026 Jan 25;16(3):379. doi: 10.3390/ani16030379 (PMC12896654; doi:10.3390/ani16030379)
Supplement: Supplementary file 1 [file animals-16-00379-s001.zip › animals-4094135-supplementary.pdf]

Dietary Fish Oil and a Flavor and Multi-Enzyme Complex Supplementation Improved the Reproductive Performance, Nutrient Metabolism and Health of Primiparous Lactating Sows and Piglets

**Lianpeng Zhao [1,†], Fangyuan Chen [2,†], Hu Zhang [1], Lingjie Huang [1], Liang Hu [3], Lun Hua [1], Lianqiang Che [1], Bin Feng [1], Yong Zhuo [1], Yan Lin [1], Shengyu Xu [1], De Wu [1], Pierre Cozannet [4], Simon Eskinazi [5] and Zhengfeng Fang [1,3,]*

[1] Key Laboratory for Animal Disease Resistance Nutrition of the Ministry of Education, Animal Nutrition Institute, Sichuan Agricultural University, Chengdu 611130, China; zlp9426@163.com (L.Z.); zhu68842@gmail.com (H.Z.); 71424@sicau.edu.cn (L.H.); hualun@sicau.edu.cn (L.H.); che.lianqiang@sicau.edu.cn (L.C.); fengbin@sicau.edu.cn (B.F.); zhuoyong@sicau.edu.cn (Y.Z.); linyan@sicau.edu.cn (Y.L.); shengyuxu@sicau.edu.cn (S.X.); wude@sicau.edu.cn (D.W.)

[2] School of Food and Liquor Engineering, Sichuan University of Science and Engineering,
Zigong 643000, China; c2023fangyuan@163.com

[3] Key Laboratory of Agricultural Product Processing and Nutrition Health (Co-Construction by Ministry and Province), College of Food Science, Sichuan Agricultural University, Ya'an 625014, China; huliang@sicau.edu.cn

[4] Adisseo France SAS, European Lab of Innovation, Science and Expertise, F-03600 Commentry, France; pierre.cozannet@adisseo.com

[5] Adisseo (Nutriad Ltd.) 1 Telford Court, Chester Gates, Chester CH1 6LT, UK; simon.eskinazi@adisseo.com

[†] These authors contributed equally to this work.

* Correspondence: zfang@sicau.edu.cn

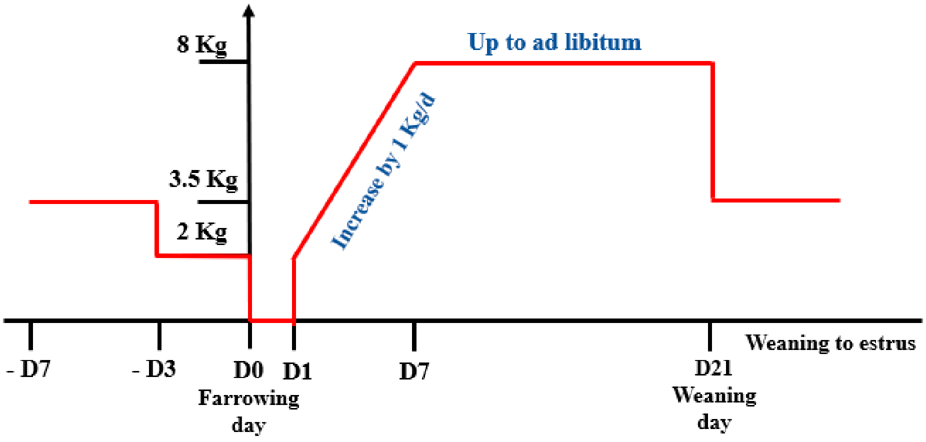

Supplementary Figure S1. The feed regimen during trial period

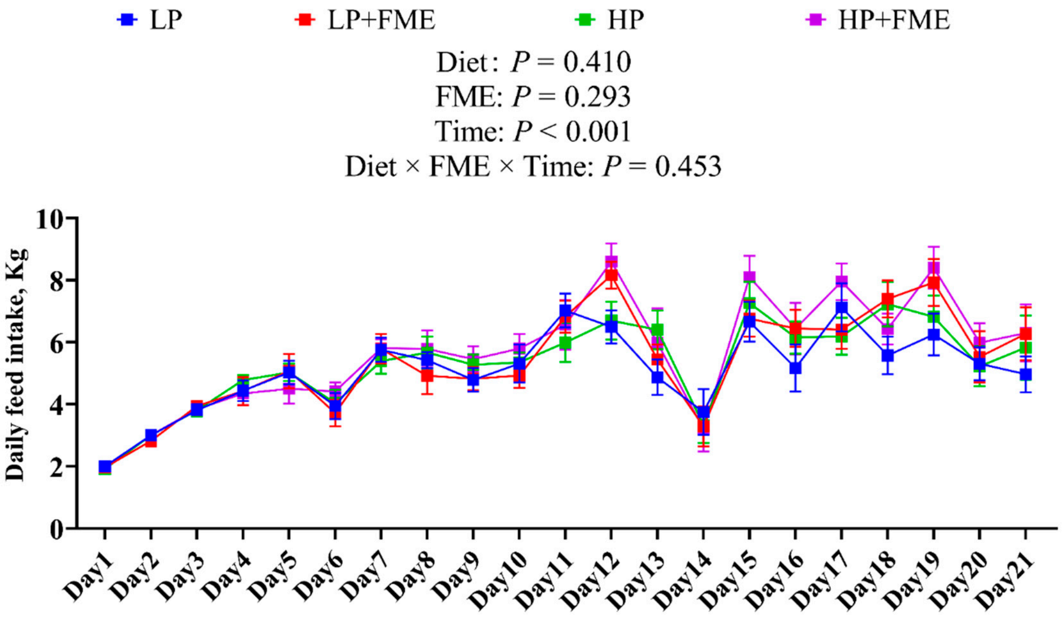

Supplementary Figure S2. Effects of PUFA levels and FME supplementation on daily feed intake of sows during lactation

Supplementary Table S1. Effects of PUFA levels and FME supplementation on the serum amino acids composition of sows

Item	Treatment				*P*-value						
	LP	LP+FME	HP	HP+FME	Diet	FME	Time	Diet× FME	Diet×Time	FME× Time	Diet× FME× Time
Essential amino acids (EAA), nmol/mL											
Lys					0.099	0.089	0.404	0.152	0.072	0.949	0.786
L0	112.56±15.70	131.88±14.80	161.78±14.04	177.09±15.70							
L7	156.92±15.70	172.90±14.80	155.08±14.04	162.86±15.70							
L15	126.96±15.70	162.85±14.80	150.82±14.82	148.20±15.70							
L22	141.53±15.70	165.53±14.80	163.88±14.80	151.78±15.70							
Met					0.102	0.466	<0.001	0.120	0.023	0.634	0.548
L0	30.04±3.34	36.10±3.15	44.13±2.99	41.34±3.34			B				
L7	44.64±3.37	44.78±3.18	51.02±3.02	43.46±3.37			A				
L15	34.82±2.18	36.28±2.05	34.98±2.05	32.27±2.18			B				
L22	44.83±2.30	42.67±2.17	45.00±2.06	43.31±2.30			A				
Trp					0.262	0.388	<0.001	0.122	0.855	0.573	0.503
L0	18.26±1.18	18.32±1.11	22.02±1.05	17.87±1.18			B				
L7	12.90±1.06	13.10±1.00	13.91±0.95	13..78±1.06			C				
L15	31.65±3.08	34.13±2.91	36.87±2.91	32.66±3.08			A				
L22	14.08±1.61	14.20±1.52	14.97±1.44	13.65±1.61			C				
Thr											
L0	63.71±5.86[ab]	70.87±5.52[a]	83.02±5.53[a]	34.97±5.86[bc]	0.554	0.006	<0.001	0.004	0.293	0.052	<.001
L7	31.13±6.21[c]	28.20±5.86[c]	36.78±5.56[bc]	28.33±6.21[c]							
L15	69.34±5.59[a]	78.48±5.27[a]	72.72±5.28[a]	66.73±5.59[a]							

L22	29.55±3.54[c]	17.80±3.33[c]	28.98±3.16[c]	24.34±3.79[c]							
Ile					0.124	0.552	0.006	0.101	0.544	0.657	0.310
L0	81.79±20.48	81.63±19.31	111.51±18.32	118.42±20.48			AB				
L7	69.26±10.07	89.70±9.49	96.95±9.01	72.30±10.07			B				
L15	95.25±12.53	115.07±11.82	127.16±11.80	95.94±12.53			A				
L22	96.32±7.73	90.02±7.29	108.53±6.92	86.38±7.73			AB				
Leu					0.060	0.778	0.019	0.076	0.473	0.898	0.201
L0	121.24±26.71	120.90±25.18	162.90±23.89	180.17±26.71			B				
L7	136.33±18.92	187.57±17.84	186.26±16.92	158.25±18.92			AB				
L15	161.33±22.47	207.25±21.19	231.75±21.21	183.64±22.47			A				
L22	181.63±18.00	180.95±16.97	191.03±16.99	179.79±18.00			AB				
Val					0.257	0.479	0.454	0.151	0.514	0.637	0.391
L0	180.83±41.84	205.12±39.45	223.34±37.42	289.09±44.69							
L7	213.41±25.37	268.78±23.92	257.25±22.69	226.57±25.37							
L15	211.50±28.43	262.40±26.80	278.65±26.78	220.26±28.43							
L22	213.34±19.53	230.85±18.41	238.46±17.47	207.56±19.53							
Phe					0.064	0.841	<0.001	0.122	0.167	0.661	0.139
L0	70.78±8.48	56.02±8.00	81.77±7.59	81.38±8.48			B				
L7	51.32±4.77	63.55±4.50	65.49±4.27	55.74±4.77			C				
L15	81.98±7.33	101.27±6.91	103.0±6.92	93.68±7.33			A				
L22	76.14±4.51	78.17±4.25	77.52±4.03	72.66±4.51			B				
His					0.520	0.416	<0.001	0.034	0.442	0.407	0.986
L0	73.58±5.31	88.15±5.01	85.47±4.75	87.36±5.31			C				
L7	99.99±6.99	110.65±6.59	111.71±6.25	110.51±6.99			A				
L15	86.36±4.66	94.43±4.39	90.05±4.34	84.11±4.66			BC				
L22	92.19±4.99	97.23±4.70	100.55±4.46	88.80±4.99			B				

Arg					0.563	0.248	0.015	0.308	0.174	0.730	0.494
L0	152.57±11.74	183.88±11.07	194.42±10.50	186.09±11.74			AB				
L7	174.17±11.74	174.78±11.07	172.94±10.50	171.98±11.74			AB				
L15	170.87±11.74	193.88±11.07	185.57±11.07	192.02±11.74			A				
L22	165.39±11.74	167.55±11.07	153.08±10.50	158.38±11.74			B				
Non-essential amino acids (NEAA) , nmol/mL											
Tyr					0.122	0.329	<0.001	0.466	0.024	0.082	0.542
L0	48.84±16.87	66.70±15.91	71.81±15.09	97.69±16.87			A				
L7	47.38±7.66	60.25±7.22	64.85±6.85	78.65±8.82			A				
L15	45.09±4.70	45.85±4.43	44.03±4.43	31.88±4.70			B				
L22	44.53±3.76	49.73±3.55	54.09±3.36	36.84±3.76			B				
Ser					0.531	0.273	<0.001	0.067	0.607	0.412	0.001
L0	68.27±8.28^{bc}	88.97±7.81^{ab}	107.82±7.41^{a}	57.39±8.86^{bc}							
L7	64.13±8.28^{bc}	61.05±7.81^{bc}	63.20±7.41^{bc}	66.56±8.28^{bc}							
L15	121.76±8.28^{a}	108.28±7.81^{a}	108.67±7.81^{a}	109.91±8.28^{a}							
L22	41.96±8.28^{c}	49.90±7.81^{c}	56.39±7.41^{bc}	54.64±8.86^{bc}							
Glu					0.537	0.114	<0.001	0.875	0.658	0.122	0.694
L0	94.18±10.54	112.75±9.94	124.16±9.43	102.90±10.53			B				
L7	144.32±13.80	132.77±13.01	151.02±12.34	131.48±13.80			A				
L15	132.21±63.45	232.22±59.82	137.72±60.26	312.49±63.45			A				
L22	156.24±15.88	157.26±15.68	149.94±14.21	149.98±16.74			A				
Gly					0.296	0.910	<0.001	0.487	0.449	0.697	0.504
L0	516.32±55.06	590.42±51.91	689.67±49.25	679.31±55.06			C				
L7	1083.64±108.61	1027.10±102.40	1030.26±97.14	1149.13±108.61			A				
L15	889.99±80.42	777.23±75.82	826.55±75.55	835.07±80.42			B				
L22	989.53±77.60	921.10±73.16	987.02±69.40	1006.16±77.60			A				

Ala					0.188	0.873	<0.001	0.509	0.303	0.783	0.062
L0	530.01±51.53	694.20±48.58	671.76±46.09	577.26±51.53			A				
L7	567.96±37.65	524.57±35.49	512.53±33.67	537.32±37.65			B				
L15	433.05±28.63	400.38±26.99	332.72±26.74	351.79±28.63			D				
L22	495.47±31.35	449.68±29.55	441.95±28.04	434.76±31.35			C				
Cys					0.580	0.678	<0.001	0.332	0.307	0.088	0.855
L0	8.12±3.45	14.73±3.25	13.62±3.08	19.22±3.45			BC				
L7	13.52±2.70	14.65±2.54	14.81±2.41	15.43±2.70			B				
L15	22.58±1.68	21.85±1.59	23.68±1.59	17.72±1.68			A				
L22	10.88±1.56	11.07±1.47	10.94±1.40	7.74±1.56			C				
Pro					0.047	0.076	<0.001	0.875	0.221	0.441	0.560
L0	162.75±32.80	214.67±30.92	254.07±29.33	273.73±32.80			B				
L7	266.78±23.77	276.00±22.41	255.59±21.26	313.56±23.77			A				
L15	187.48±15.00	216.30±14.15	195.17±14.15	208.59±15.00			B				
L22	197.64±14.38	201.92±13.55	212.97±12.86	202.93±14.38			B				
Other AAs, nmol/mL											
Cit					0.214	0.287	0.007	<0.001	0.516	0.922	0.439
L0	66.71±5.87	81.60±5.53	85.35±5.25	63.00±5.87			A				
L7	67.13±5.87	72.88±5.53	61.88±5.25	56.59±5.87			AB				
L15	62.57±5.87	70.33±5.53	74.47±5.53	56.59±5.87			AB				
L22	57.60±5.87	65.67±5.53	66.75±5.25	51.62±5.87			B				
Tau					0.057	0.706	<0.001	0.462	0.326	0.583	0.564
L0	92.21±12.00	112.07±11.32	139.35±10.73	114.22±12.00			C				
L7	175.78±15.83	184.48±14.92	200.93±14.16	220.71±15.83			A				
L15	151.84±14.28	164.32±13.56	163.50±13.28	164.40±14.38			B				
L22	189.47±12.58	186.65±11.86	199.86±11.25	189.90±12.58			A				

Orn					0.089	0.047	<0.001	0.593	0.271	0.659	0.444
L0	44.55±11.55	56.02±10.89	64.13±10.33	82.16±11.55			AB				
L7	64.22±7.22	68.88±6.80	57.87±6.46	65.38±7.22			A				
L15	54.53±5.85	62.95±5.52	60.65±5.55	61.80±5.85			A				
L22	42.13±3.50	52.23±3.30	53.73±3.13	46.84±3.50			B				
3-Mehis					0.245	0.059	<0.001	0.934	0.780	0.659	0.180
L0	17.25±3.43	17.18±3.23	17.79±3.07	20.85±3.43			B				
L7	26.53±5.31	27.42±5.01	23.75±4.75	37.63±5.31			A				
L15	23.36±2.58	30.45±2.44	29.72±2.44	27.88±2.58			A				
L22	18.81±1.66	22.67±1.56	21.59±1.48	19.33±1.66			B				
Amino acid groups, nmol/mL											
Total EAA					0.077	0.713	0.071	0.015	0.428	0.784	0.637
L0	905.34±106.82	992.87±100.71	1162.04±95.55	1177.65±106.82							
L7	990.08±83.78	1154.02±78.99	1147.37±74.94	1043.79±83.78							
L15	1070.04±92.52	1286.05±87.23	1312.22±87.49	1149.51±92.52							
L22	1055.01±67.40	1084.97±63.54	1143.52±63.55	1023.62±67.40							
Total NEAA					0.369	0.698	<0.001	0.985	0.404	0.665	0.217
L0	1428.49±112.00	1782.43±105.60	1932.90±100.18	1800.32±112.00			B				
L7	2187.71±161.43	2096.38±152.20	2094.24±144.39	2267.96±161.43			A				
L15	1832.16±141.66	1802.12±133.56	1671.53±132.21	1867.44±141.66			B				
L22	1936.26±122.30	1823.18±115.31	1913.28±109.39	1867.48±122.30			B				
BCAA					0.103	0.888	0.307	0.037	0.653	0.773	0.404
L0	383.85±84.17	407.65±79.36	497.75±75.29	551.55±84.17							
L7	419.01±51.69	546.05±48.73	540.45±46.23	457.13±51.69							
L15	468.08±62.31	584.72±58.74	638.20±58.91	499.84±62.31							
L22	491.29±41.66	501.82±39.28	549.60±39.29	473.74±41.66							

Total AAs					0.067	0.420	0.003	0.183	0.111	0.541	0.591
L0	2554.56±171.96	3042.17±162.13	3401.55±153.81	3258.21±171.96			B				
L7	3511.44±191.49	3604.07±180.54	3586.02±171.28	3692.06±191.49			A				
L15	3194.49±132.77	3416.22±125.18	3312.08±124.92	3328.62±132.77			B				
L22	3299.27±146.55	3235.37±138.16	3332.10±138.19	3198.79±146.55			B				

L0 = Day 0 of lactation; L7 = Day 7 of lactation; L15 = Day 15 of lactation; L22 = Day 22 of lactation.

[a, b, c] (Tukey's post-hoc test following significant Diet × FME × Time interaction) or A, B, C (Time) means within a row with no common letters differ at $P < 0.05$ or $P < 0.01$.

Supplementary Table S2. Effects of PUFA levels and FME supplementation on the serum amino acids composition of piglets

Item	Treatment				*P*-value						
	LP	LP+FME	HP	HP+FME	Diet	FME	Time	Diet× FME	Diet×Time	FME× Time	Diet× FME× Time
Essential amino acids (EAA), nmol/mL											
Lys					0.925	0.778	0.011	0.903	0.422	0.536	0.650
PD0	206.83±68.82	257.47±60.46	203.21±57.36	214.56±64.13			B				
PD22	305.83±24.71	284.00±23.29	325.04±22.10	325.03±24.71			A				
Met					0.070	0.548	<0.001	0.292	0.424	0.915	0.920
PD0	68.01±21.68	49.23±20.44	24.87±19.39	32.38±21.68			B				
PD22	101.51±8.12	82.60±7.65	78.44±7.26	81.36±8.12			A				
Trp					0.512	0.309	<0.001	0.051	0.347	0.516	0.341
PD0	25.82±3.35	21.83±3.16	18.36±2.99	20.27±3.35			B				
PD22	59.29±4.74	46.23±4.47	51.77±4.24	55.41±4.74			A				
Thr					0.193	0.529	<0.001	0.473	0.591	0.139	0.780
PD0	65.19±17.19	27.57±16.21	31.43±16.26	17.46±18.45			B				
PD22	141.19±17.19	147.10±16.21	127.11±15.37	143.23±17.19			A				
Ile					0.778	0.823	<0.001	0.760	0.095	0.828	0.902
PD0	70.52±26.64	62.45±23.23	43.97±22.04	41.01±24.64			B				
PD22	109.92±7.76	104.00±7.31	120.96±6.94	126.08±7.76			A				
Leu					0.303	0.750	0.084	0.971	0.166	0.833	0.970
PD0	213.94±67.00	196.28±63.17	132.44±59.93	114.69±67.00							
PD22	220.24±16.03	214.08±15.12	229.76±14.34	228.54±16.03							
Val					0.236	0.912	0.469	0.553	0.072	0.988	0.531

PD0	354.28±60.50	319.73±57.04	223.43±54.11	264.11±60.50							
PD22	301.95±19.63	305.55±18.50	317.93±19.56	322.28±19.63							
Phe					0.112	0.823	<0.001	0.453	0.203	0.847	0.765
PD0	105.77±25.72	86.60±24.25	53.99±23.01	62.25±25.72			B				
PD22	158.27±8.34	152.15±7.86	148.56±7.46	154.03±8.34			A				
His					0.087	0.307	<0.001	0.440	0.202	0.760	0.807
PD0	114.74±21.50	92.53±20.27	63.90±19.12	67.33±21.50			B				
PD22	153.94±14.06	130.17±13.25	140.25±12.57	130.37±14.06			A				
Arg					0.276	0.407	<0.001	0.361	0.526	0.937	0.765
PD0	75.94±35.95	107.73±31.58	79.77±29.96	83.08±33.50			B				
PD22	282.56±35.55	332.85±33.52	270.12±31.80	262.74±35.55			A				
Non-essential amino acids (NEAA)											
Try					0.074	0.976	0.019	0.138	0.177	0.845	0.468
PD0	191.72±45.17	135.87±42.58	68.46±40.40	114.11±45.17			B				
PD22	195.38±12.10	181.23±11.41	166.77±10.82	188.40±12.10			A				
Ser					0.697	0.805	<0.001	0.945	0.287	0.221	0.520
PD0	112.54±15.93	98.60±14.02	98.58±13.30	67.84±14.87			B				
PD22	317.91±29.24	321.22±27.57	316.38±26.16	341.01±29.24			A				
Glu					0.243	0.755	0.157	0.433	0.609	0.237	0.297
PD0	158.93±85.93	103.77±81.01	76.02±81.22	255.26±85.93							
PD22	229.82±95.74	155.60±90.26	356.49±85.93	235.48±95.74							
Gly					0.489	0.923	0.001	0.100	0.858	0.382	0.114
PD0	1160.63±128.09	971.85±120.77	961.85±114.57	1255.09±128.09			B				
PD22	1345.65±80.72	1257.50±76.11	1391.58±72.20	1344.23±80.72			A				
Ala					0.156	0.403	<0.001	0.429	0.697	0.408	0.643
PD0	1325.49±148.80	1096.70±140.29	1085.06±133.09	1050.66±148.80			A				

PD22	891.77±60.66	858.63±57.19	777.24±54.25	801.21±60.66			B				
Cys					0.484	0.766	<0.001	0.141	0.788	0.515	0.239
PD0	45.09±5.65	32.35±5.33	32.58±5.06	38.10±5.65			A				
PD22	21.00±4.56	22.57±4.30	19.82±4.08	21.47±4.56			B				
Pro					0.891	0.717	<0.001	0.780	0.659	0.412	0.717
PD0	253.95±103.54	360.98±83.89	257.85±79.58	289.84±88.98			B				
PD22	772.13±74.92	738.23±70.64	784.92±67.01	762.32±74.92			A				
Other AAs											
Cit					0.385	0.888	0.081	0.687	0.232	0.706	0.574
PD0	121.82±16.62	107.87±15.67	93.09±14.86	97.33±16.62							
PD22	118.50±10.24	121.97±9.65	122.61±9.16	123.41±10.24							
a-ABA					0.126	0.341	<0.001	0.451	0.959	0.976	0.753
PD0	6.30±5.33	5.72±5.02	8.07±4.77	15.00±5.33			B				
PD22	30.47±5.06	32.43±4.77	34.19±4.53	39.02±5.06			A				
Tau					0.752	0.779	<0.001	0.945	0.177	0.523	0.703
PD0	149.63±19.54	141.95±18.42	160.46±17.47	160.33±19.54			B				
PD22	358.18±22.37	379.22±21.09	341.22±20.00	348.39±22.37			A				
Orn					0.443	0.723	<0.001	0.387	0.092	0.817	0.237
PD0	137.49±22.91	103.33±21.60	79.83±20.49	98.51±22.91			B				
PD22	193.13±14.10	192.65±13.29	204.38±12.61	200.27±14.10			A				
3-Mehis					0.021	0.916	0.134	0.879	0.298	0.576	0.104
PD0	11.85±2.34	10.43±2.21	4.28±2.10	7.76±2.51							
PD22	10.84±2.03	12.98±1.91	12.05±1.81	8.38±2.03							
Amino acid groups											
Total EAA					0.617	0.638	<0.001	0.832	0.588	0.583	0.681
PD0	975.64±290.42	1221.43±255.14	917.08±255.93	969.96±290.42			B				

PD22	1834.59±108.54	1798.73±102.33	1810.02±97.08	1829.06±108.54			A				
Total NEAA					0.755	0.999	<0.001	0.428	0.844	0.367	0.631
PD0	2871.26±326.59	2800.12±286.87	2642.98±287.80	3071.19±304.27			B				
PD22	3773.64±253.52	3234.98±239.02	3813.20±253.52	3694.11±253.52			A				
BCAA					0.315	0.894	0.056	0.750	0.096	0.889	0.824
PD0	638.74±147.29	578.67±138.86	399.83±131.74	419.81±147.29							
PD22	632.01±39.39	623.63±37.14	668.64±35.23	676.89±39.39							
Total AAs					0.884	0.800	<0.001	0.713	0.910	0.430	0.867
PD0	4209.21±609.52	4385.13±535.43	3591.06±571.50	4524.45±609.52			B				
PD22	6288.88±362.42	6040.53±341.69	6303.47±324.16	6203.63±362.42			A				

PD0: piglets on day 0 of lactation; PD22: piglets on day 22 of lactation. A, B (Time) means within a row with no common letters differ at $P < 0.05$ or $P < 0.01$.
